# Physical Examination of Potential Deceased Organ and Tissue Donors: An Overview of the European Landscape

**DOI:** 10.3389/ti.2023.11394

**Published:** 2023-07-21

**Authors:** Akila Chandrasekar, Richard Lomas, Jacinto Sánchez-Ibáñez, Mar Lomero, Arlinke Bokhorst, Margarida Ivo Da Silva, Esteve Trias, Alicia Pérez Blanco, Beatriz Domínguez-Gil, Marta López-Fraga

**Affiliations:** ^1^ Tissue and Eye Services, NHS Blood and Transplant, Liverpool, United Kingdom; ^2^ Tissue Establishment and Cryobiology unit, A Coruña University Hospital, Coruña, Spain; ^3^ European Directorate for the Quality of Medicines and Healthcare (EDQM), Strasbourg, France; ^4^ National Office for Hemovigilance and Biovigilance (TRIP), Leiden, Netherlands; ^5^ National Coordination for Transplantation, Instituto Portugues do Sangue e da Transplantacao, Lisbon, Portugal; ^6^ Hospital Clinic of Barcelona, Barcelona, Spain; ^7^ Leitat Technological Center, Barcelona, Spain; ^8^ Organización Nacional de Trasplantes, Ministerio de Sanidad, Madrid, Spain

**Keywords:** organ donation, donor assessment, donor screening, physical examination, tissue and cornea donation

## Abstract

Physical examination (PE) of donors is essential to identify potential risks to the safety and efficacy of donated organs and tissues and is mandatory in the EU. However, no detailed guidance is available as to how PE should be performed. Health authorities (HA) and health professionals (HP) in member states of the European Committee on Organ Transplantation of the Council of Europe (CD-P-TO) and observer countries completed surveys relating to the regulatory requirements for PE and the professional practice of PE in their countries for organ and tissue donors. The HA survey addressed regulatory aspects, and the HP survey addressed professional practices, training, and respondents’ opinions on the value of PE. These surveys revealed significant inter-country variation in the regulatory approach to PE and the performance of PE by professionals. Most respondents opined that PE was important and yielded valuable information in identifying contraindications to donation. There is no consensus at a regulatory or professional level as to how PE should be performed on organ and tissue donors. There is a requirement for agreed best practice guidelines in this area.

## Graphical Abstract

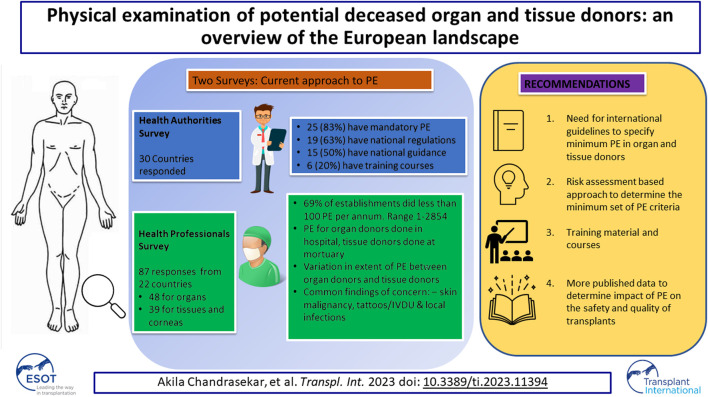

## Introduction

Organizations/establishments that are active in the field of tissue and organ procurement from deceased donors perform a comprehensive donor assessment to mitigate the risk of transmission of infection and disease from donors to recipients and to optimize the quality and safety of donated material in order to maximize the probability of good clinical outcomes. Physical examination (PE) is used in conjunction with review of medical records, medical history obtained from referring professionals, interviews with donor families, information from general practitioners, autopsy reports (if applicable), and screening tests, as an essential part of this comprehensive donor evaluation. It should be noted that PE performed to evaluate the suitability of an individual to donate organs and tissues differs significantly from PE performed on a living individual during a standard medical examination. When considering donation, PE focusses on indications that relate specifically to the safety and quality of donated material rather than indicators of a patient’s health.

Directive 2006/17/EC (technical requirements for the donation, procurement and testing of human tissues and cells) [1] states in Annex I “Selection criteria for donors are based on an analysis of the risks related to the application of the specific cells/tissues. Indicators of these risks must be identified by physical examination … ,” and Annex IV states “…in the case of a deceased donor […] a physical examination of the body must be performed to detect any signs that may be sufficient in themselves to exclude the donor, or which must be assessed in the light of the donor’s medical and personal history.” and that these findings must be recorded. Similarly, Directive 2010/45/EU (standards of quality and safety of human organs intended for transplantation) [2] states “Information from a potential donor’s medical history, physical examination and complementary tests should be collected for the adequate characterization of the organ and the donor.”

Thus, at EU level all donor coordinators, organ procurement organizations and tissue establishments (TEs) adhering to these requirements are obliged to perform a documented PE prior to procurement. However, neither directive specifies the content of the PE, who should perform the PE, or how it should be performed and documented. The focus of the PE of a potential tissue or organ donor differs from the medical examination performed on the same individual during admission to hospital as a patient. PE should therefore be performed in all cases of donation of tissues or organs in order to systematically identify evidence of infections or diseases that could be transmitted through organs, tissues and corneas and pose a risk to the transplant recipient, as well as to better assess the quality of the donated substance [3]. The findings of PE complement the comprehensive clinical data collected on each potential donor [4].

The EDQM “Guide to the quality and safety of organs for transplantation” [5] and “Guide to the quality and safety of tissues and cells for human application” [6] provide basic guidance on what to look for in the PE of deceased organ and tissue donors. In general, the objective of PE is to identify physical manifestations of disorders that could be an indication for a condition listed in the exclusion criteria for donation. There are, however, only a very limited number of studies [7] that have evaluated what PE should consist of, how it should be performed, and the added value of physical findings noted in relation to the final donor evaluation.

For this reason, the European Committee on Organ Transplantation of the Council of Europe (CD-P-TO)^1^ conducted a survey to determine the current practices for performing PE and the regulatory approach in Council of Europe (CoE) member states, with a view to developing guidance on best practice.

## Methods

Two different English language survey questionnaires were prepared: one to investigate the regulatory framework (legally binding and non-legally binding documents) governing the PE of organ and tissue donors to be completed by health authorities (HAs) and the second to capture the actual practices of health professionals (HPs) performing PE of organ and tissue donors. A CD-P-TO working group was set up to develop and validate the questions for the surveys. Both final survey questionnaires were piloted in a limited number of member states, using CD-P-TO representatives as contact points, to evaluate their content and the use of English language terminology prior to wider circulation. The final surveys were circulated to member states via their CD-P-TO representatives, who disseminated them nationally. Responses were gathered electronically using an online survey tool (Surveymonkey.com). Prior to analysis, all responses were reviewed to remove any invalid responses—for example, instances where respondents had submitted a partial response prior to providing a full response at a later date. In total, five incomplete responses from the HP survey were removed.

The HA survey consisted of 10 questions (Q), Q1–Q5 to gather country-specific general/demographic information and Q6–Q10 to collect information on regulations in place related to the practice of PE (Supplementary Datasheet S1). The HP survey contained 35 questions divided into five sections. The first 6 (Q1–Q6) concerned respondents’ profiles and the next 10 (Q7–Q16) were related to their organization, followed by questions relating to their practices for performing PE (Q17–Q25), training (Q26–Q31) and a final section on their personal views about the value of PE (Q32–35). A distinction was made between responses from those who perform PE on organ donors, multi-tissue donors and cornea donors because of the differences in donor selection criteria. The same HP questionnaire was used in all cases, but a separate response was requested for each type of donor (Supplementary Datasheet S2). Some organizations that responded to the HP survey were responsible for PE for different types of donors. In these cases, the organization was asked to submit a separate response for each type of donor.

## Results

The surveys were distributed among representatives in CD-P-TO member [37] and observer [5] countries. Seven member countries (Albania, Latvia, Malta, North Macedonia, Norway, Turkiye and Ukraine) and one observer country (United States) did not respond to either the HA or the HP survey, and one observer country (Armenia) was excluded from the analysis after responding that they did not currently have a deceased donor program. In total, 33 of 42 countries (79%) responded to one or both of the surveys as shown in Table 1.

**TABLE 1:** CD-P-TO PE survey responses.

Country	HA	Organ	Tissue	Cornea
Austria	1	2	2	2
Belgium	1	1		
Bulgaria	1	1	1	1
Croatia	1	2		
Cyprus	1			
Czech Republic		3		
Denmark	1			
Estonia	1	2		
Finland		2		
France	1	1		1
Germany	1	0	1	2
Greece		1		
Hungary	1	2		1
Ireland	1			
Italy	1	5	3	4
Lithuania	1	2		
Luxembourg	1			1
Moldova	1	1	2	
Montenegro	1			
Netherlands	1			
Poland	1	1		
Portugal	1	7	1	4
Romania	1			
Serbia	1	1		
Slovak Republic	1	3	1	
Slovenia	1			
Spain	1	4	2	4
Sweden	1	1	1	1
Switzerland	1	3		1
United Kingdom	1	3	2	1
Canada	1			
Georgia	1			
Israel	1			
Total	30	48	16	23

Multiple responses to the HP survey were received from some countries, as discussed below.

### HA Survey

Thirty responses (70% response rate) were received for the HA survey (Table 1); 83% of respondents, representing 25 countries, declared that PE is mandatory in their countries for either organs or tissues, or for both. However, only 63% (19 countries) have national regulations related to PE. Fifteen of the 30 respondents (50%) have national guidance documents related to PE; however, 55% (16 countries) reported that they do not have a uniform template/model (form) in their country to record the findings of the PE. The majority of respondents (21 countries, 70%) noted that they had no specific training course covering aspects related to the PE of tissue donors.

### HP Survey

**FIGURE 1:**
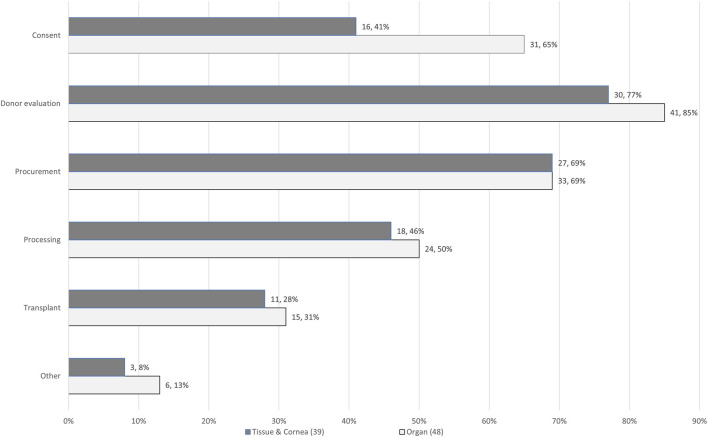
Roles of HPs (87 respondents) completing the survey.

There were 87 responses from 22 countries for the HP survey, 48 related to organ donors, 16 to tissue donors, and 23 to cornea donors, as shown in Table 1. More than half of respondents (46, 54%) identified themselves as donor/transplant coordinators, 19 (22%) as medical directors/assistant directors/Responsible Persons, 11 (13%) as transplant surgeons, 9 (11%) had other job titles, such as retrieval team leader or member, and 2 did not provide their job title. They had various roles in the organ or tissue donation and transplantation pathway as shown in Figure 1 (some had multiple roles).

The survey included responses from organizations/establishments involved in one or more activities. Some organizations (29, 33%) had responsibilities for both organ/tissue procurement and for processing and banking as a TE. In total, 119 responses were received from 87 individual respondents, with 71 (82%) from organ/tissue procurement organizations and hospitals responsible for donor consent, medical history, and procurement, 42 (48%) from TEs responsible for procurement, processing, storage, and distribution and 6 (7%) from TEs who have agreements with external organizations to perform procurement. These organizations/establishments facilitated between 1 and 2,854 donations in one calendar year. These responses were categorized into establishments performing <100 PEs, 101–500 PEs and >501 PEs (Table 2). The majority (69%) of responding establishments performed <100 PEs per year. PE of organ donors was always (100%) done in a hospital setting, whereas PE of tissue donors was mainly (74%) done in a mortuary setting. All of the PEs of organ donors and most of those of tissue and cornea donors are done by those with medical or nursing qualifications. A small proportion (14%) of PE of tissue and cornea donors are done by individuals with non-medical/nursing qualifications (Table 2).

**TABLE 2:** Details of activity in the organizations/establishments.

		Organ (O) (48)	Tissue/Cornea (TC) (39)
When is PE routinely performed in the organization/establishment?	During/after donor medical assessment	46 (96%)	12 (31%)
	Prior to procurement (after refrigeration)	0	25 (64%)
	Not performed in our establishment	2 (4%)	2 (5%)
Number of deceased donor PEs performed in 2019 in the organization/establishment (Range: 1–2,854) No response (6): O:3 + TC:3	Low (1–100)	37 (82%)	19 (53%)
	Medium (101–500)	4 (9%)	12 (33%)
	High (501 and above)	4 (9%)	5 (14%)
Number of HPs who performed PEs in the organization/establishment in 2019 (Range: 1–320) No answer (12): O:6 + TC:6	Low (1–10)	22 (52%)	22 (67%)
	Medium (11–100)	15 (36%)	10 (30%)
	High (101 and above)	5 (12%)	1 (3%)
Number of PEs performed in 2019 by HPs completing the survey questionnaire (Range: 0–630) No answer (2) O:1 + TC:1	Not performed	9 (19%)	16 (42%)
	1–100	36 (77%)	18 (47%)
	Above 101	2 (4%)	4 (11%)
Describe the setting where the donor PE is performed	Hospital setting (ICU/operating theatre)	48 (100%)	19 (50%)
No answer (1) TC:1	Hospital mortuary	11 (23%)	28 (74%)
	Forensic department	1 (2%)	9 (24%)
	Other	1 (2%)	4 (10%)
Who performs the donor PE in your establishment?	HP in charge of the donor (GP, hospital physician, nurse, etc.)	32 (67%)	12 (32%)
	Organ or tissue coordinator	34 (71%)	18 (47%)
	Professional from the procurement team of the TE	12 (25%)	24 (63%)
	Pathologist/forensic examiner	6 (13%)	8 (21%)
	Other	0	1 (3%)
Basic qualifications of the HP performing the donor PE	Medical	32 (67%)	26 (68%)
No answer (1) TC:1	Nursing	16 (33%)	7 (18%)
	Graduate (e.g., science degree) or similar professional qualifications	0	3 (8%)
	Other (please specify)	0	2 (6%)
Does your establishment/organization use total body CT scan as a routine examination for tissue/organ donors? No answer (1) TC:1	Yes, always	4 (8%)	0
	Yes, in selected cases	21 (44%)	4 (11%)
	No, not used	23 (48%)	34 (89%)
Time to complete PE	Less than 5 min	2 (5%)	7 (20%)
No answer (10) O:5, TC:5	5–15 min	18 (42%)	18 (53%)
	16–30 min	14 (32%)	5 (15%)
	31–60 min	6 (14%)	2 (6%)
	More than 60 min	3 (7%)	2 (6%)

In 2019, 60 (71%) HPs who responded had performed one or more PEs and 25 (29%) had not performed any; 2 respondents did not answer this question (Table 2). Four respondents (8%) reported that total body CT scan is routinely performed as part of the PE of organ donors and 21 (44%) that is done in selected cases, for example, donors aged over 50 and donors with suspected malignancy. Routine use of CT scan was not reported in any tissue or cornea donor responses, with only 11% reporting use in selected cases (Table 2).

### Carrying Out PE

The most common response for time taken to complete the PE was 5–15 min, reported in 42% of responses for organ donors and 53% of responses for tissue and cornea donors (Table 2). There were differences between organ, tissue, and cornea donors in terms of the number of persons present (Table 3), techniques used (Table 4) and the PE process (Table 5). The responses in the HA survey were compared with those of the HP survey to determine whether there is a variation in PE practice existing between countries with and without national guidelines. Using lymph node palpation in organ donors as a comparator, in countries with guidelines, 63% of responses reported that they always palpated lymph nodes and 33% reported that they sometimes did this, compared to 42% and 33% in countries without national guidelines.

**TABLE 3:** No of people present to perform PE on an individual donor.

	1	2	3	>3	Total responses	No response
Organ	10 (23%)	22 (51%)	5 (12%)	6 (14%)	43	5
Tissue	6 (43%)	7 (50%)	0 (0%)	1 (7%)	14	2
Cornea	11 (58%)	7 (37%)	0 (0%)	1 (5%)	19	4
Total	27 (36%)	36 (48%)	5 (7%)	7 (9%)	76	11

**TABLE 4:** Techniques used in performing PE.

	Organ donors (44)		Tissue/cornea donors (36)	
	Yes	No (or NA)	Yes	No (or NA)
Observation	43 (98%)	1 (2%)	36 (100%)	0
Auscultation	27 (64%)	15 (36%)	2 (6%)	31 (94%)
Palpation	37 (88%)	5 (12%)	19 (58%)	14 (42%)
Percussion	17 (41%)	24 (59%)	3 (10%)	30 (90%)

**TABLE 5:** PE Process: When donor PE is performed, do you?

	Organ (46)				Tissue/Cornea (35)			
	Always	Sometimes	Never	Total	Always	Sometimes	Never	Total
Open and examine the oral cavity	21	15	6	42	7	12	13	32
	50%	36%	14%		22%	38%	40%	
Inspect/examine the genital area	31	9	1	41	21	5	7	33
	76%	22%	2%		64%	15%	21%	
Turn the donor to examine the back	28	12	1	41	16	8	10	34
	68%	29%	3%		47%	24%	29%	
Palpate the lymph nodes	23	13	5	41	8	6	18	32
	56%	32%	12%		25%	19%	56%	
Palpate the breast tissue	23	11	6	40	7	6	19	32
	58%	27%	15%		22%	19%	59%	
Palpate the abdomen	28	6	8	42	7	8	17	32
	67%	14%	19%		22%	25%	53%	
Check for evidence of intravenous drug use	40	1	0	41	33	1	1	35
	98%	2%			94%	3%	3%	

**FIGURE 2:**
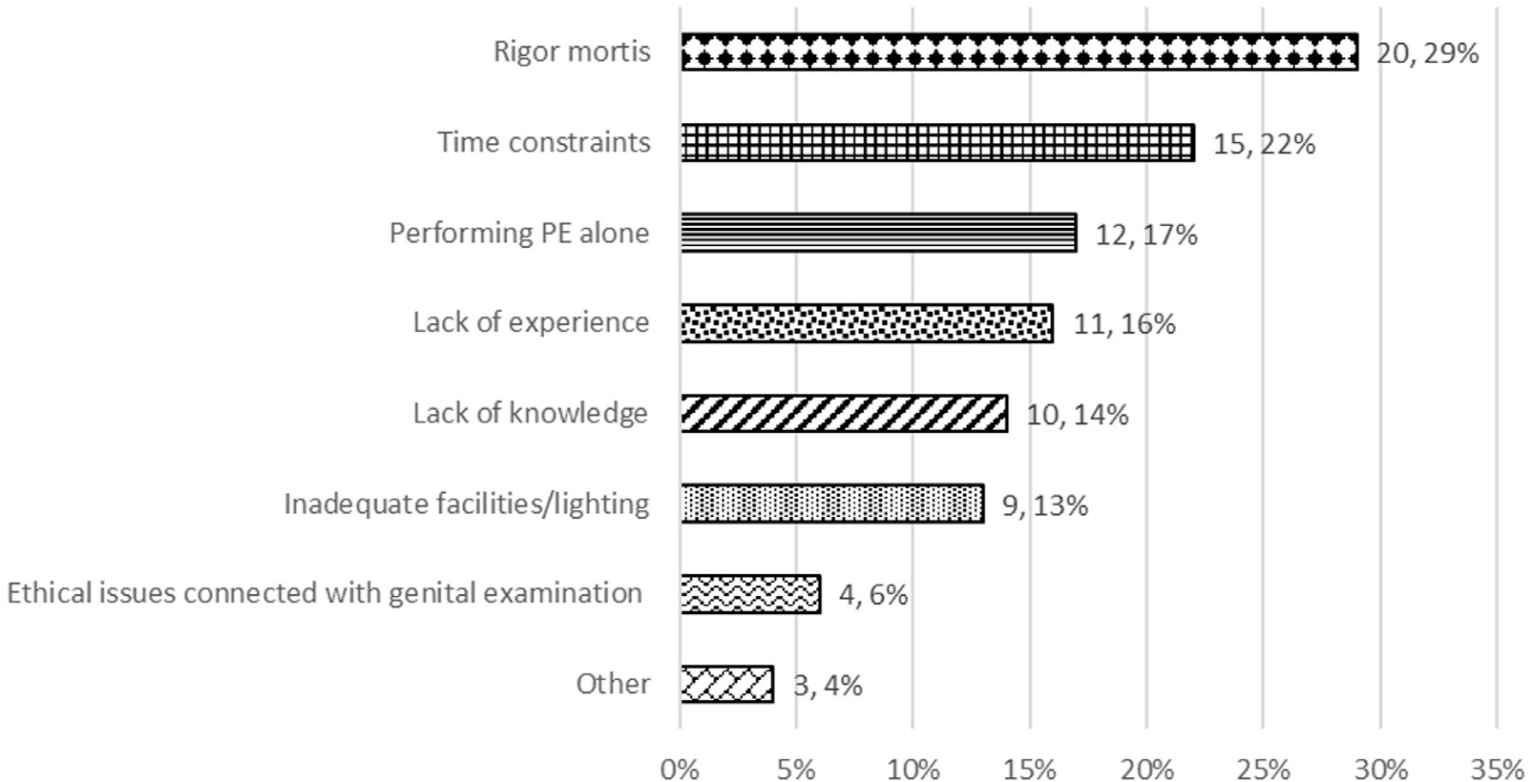
For deceased tissue and cornea donors only: common issues in your practice that are barriers to performing a detailed PE.

For cornea donors, in 58% of responses PE was done by a single person, in comparison to 43% for tissue donors and 23% for organ donors. For tissue and cornea donors, visual inspection was always done as part of the donor PE. Auscultation and percussion are not applicable because they are not possible in deceased tissue and cornea donors unless done during organ donation assessment prior to death. In total, 58% (25/43) of respondents for organs and 79% (29/36) of tissue and cornea respondents reported identifying anomalies during PE that prevented donation from proceeding at that point, or that had resulted in subsequent rejection of the procured organs/tissues. Evidence of suspected malignancy—the three most common being melanoma, abnormal lymph nodes, and breast lesion—was the main reason that prevented organ donation, skin lesion/tattoo/evidence of IV drug use were the main reasons that prevented tissue donation and corneal infection/scar/ulcer were the main reasons that prevented cornea donation. Options available for escalation should an abnormal finding be detected are shown in Table 6. Practical issues that form barriers to performing a detailed PE of deceased tissue and cornea donors are shown in Figure 2, the most commonly reported being rigor mortis.

**TABLE 6:** What options are available to you in your practice if you identify an abnormal finding?

	Organ (43)		Tissue (14)		Cornea (21)		Tissue and cornea (35)	
	No.	%	No.	%	No.	%	No.	%
Document the findings and proceed/stop	33	77	14	100	19	90	33	94
Ask a colleague to examine the donor for a second opinion	33	77	8	57	7	29	15	43
Phone a senior colleague from your team and describe your findings to obtain advice	26	60	9	64	11	52	20	57
Take a photograph and send it to an external expert (e.g., skin specialist)	23	53	4	29	7	33	11	31
Take a biopsy for histopathology examination	31	72	8	57	4	19	12	34
Other tests or non-invasive examinations (CT, MRI, Xray)	35	81	1	7	2	10	3	9
Review medical notes and/or contact general practitioner	33	77	10	71	11	52	21	60
Other (please provide details)	5	12	2	14	1	5	3	9

### Training

Almost all (82 of 87) respondents answered the question relating to training; 52 (63%) reported that they had received some kind of specific training in how to perform a donor PE. In this group, the training was primarily “on the job” practical training delivered by colleagues. Other respondents reported that training was provided by external bodies from outside of their organization. Training was delivered mainly by practical simulation, reading the SOP and/or visual presentations. Most respondents considered their training as either “very” or “extremely” valuable (Table 7).

**TABLE 7:** Training.

		Organ (O)	Tissue/Cornea (TC)
Have you received any specific training in how to perform a donor PE for organ/tissue/cornea donors?	Yes	26 (58%)	26 (70%)
No response (5): O:3 + TC:2	No	19 (42%)	11 (30%)
If you have received training, when did this take place	During my degree studies	9 (30%)	5 (18%)
No response (29): O:18 + TC:11	Provided by external bodies outside the organization	11 (37%)	10 (36%)
	Before starting to work in my establishment (during induction, including theory)	6 (20%)	9 (32%)
	Case-by-case training by another colleague during my working practice	20 (67%)	16 (57%)
	Other	4 (13%)	3 (11%)
How was the training delivered?	Reading the SOP	14 (47%)	16 (59%)
No answer (30): O:18 + TC:12	PowerPoint presentation	12 (40%)	15 (56%)
	eLearning course	6 (20%)	7 (26%)
	Practical simulation	24 (80%)	21 (78%)
	Other	4 (13%)	4 (15%)
Did the training include how to document PE findings?	Yes	17 (57%)	22 (81%)
No answer (30): O:18 + TC:12	No	13 (43%)	5 (19%)
Describe the value of the training for your daily work?	Extremely valuable	14 (47%)	16 (62%)
No answer (31): O:18 + TC:13	Very valuable	9 (30%)	7 (27%)
	Moderately valuable	4 (13%)	3 (11%)
	Slightly valuable	1 (3%)	
	Not valuable at all	2 (7%)	
Competency	Training updates (How often? Please provide details)	11 (67%)	19 (73%)
No answer (31): O:18 + TC:13	Audit	9 (30%)	7 (27%)
	Peer-review practice	13 (43%)	11 (42%)
	Task-based training using SOPs	10 (33%)	10 (38%)
	Other	5 (17%)	

### General Opinion

The views of the respondents on the value of and reasons for PE are shown in Tables 8–10. Opinions varied depending on the type of donor that was evaluated: 16% of HPs performing PEs of tissue or cornea donors considered that PE was not valuable or slightly valuable, compared to only 2% of HPs performing PEs of organ donors.

**TABLE 8:** Opinion of respondents on the value of the PE in the evaluation of deceased donors.

	Organ (43)		Tissue (15)		Cornea (22)		Tissue and cornea (37)	
	Number	%	Number	%	Number	%	Number	%
Extremely valuable	17	40%	10	67%	6	27%	16	43
Very valuable	17	40%	3	20%	7	32%	10	27
Moderately valuable	8	18%	0		5	23%	5	14
Slightly valuable	1	2%	1	7%	3	14%	4	11
Not valuable at all	0		1	7%	1	5%	2	5%

**TABLE 9:** Top 3 most important reasons selected for doing a PE prior to organ and tissue/cornea donation.

	Organ (43)		Tissue and cornea (37)	
	No.	%	No.	%
To identify the cause of death	13	30	5	14
To identify potential medical contraindications	39	91	29	78
To exclude high-risk individuals (e.g., social risks)	34	79	31	84
To confirm information available from other sources	24	56	18	49
To comply with regulations and guidelines	12	28	19	51
Transplant centers are interested in the donor PE	9	21	1	3
Not important, as the PE is of limited value for tissue donors, including cornea donors	0		4	11%

**TABLE 10:** On a scale of 1–10, with 1 indicating no importance and 10 indicating extreme importance, what is the value of abnormal findings in the donor PE in prevention of donor recipient disease transmission (safety) or graft quality?

	Mean score
Tissue/Cornea donors: Donor-recipient transmission	7.4 (29)
Tissue/Cornea donors: Graft Quality	6.3 (30)
Organ donors: Donor-recipient transmission	9.0 (27)
Organ donors: Graft Quality	6.2 (25)

Figures given as mean score with number of responses in brackets.

## Discussion

There are very few published articles relating to PE of deceased donors [7], and this is the first multi-national survey to date that has explored current practices for performing PE of potential organ, tissue, and cornea donors, soliciting feedback from both HAs and HPs. Responses to the HA survey showed that, while PE is mandatory in the majority (83%) of countries, many respondents reported that there were no nationally mandated standards or protocols for performing PE. This indicates that the performance of PE could vary between establishments and that the outcome of the donor selection process may differ within the same country. It was also evident from responses to the HP survey that there was intra-country variation in the practices for performing PE on organ and tissue donors, however, there are insufficient individual responses from different countries to draw firm conclusions in this area. Without established protocols or guidelines it is also difficult for HAs to assess whether organs or tissues meet quality standards. In order to safeguard donor selection outcomes, it is therefore necessary to establish uniform protocols for PE for different types of donors. An international body, such as the EDQM in collaboration with HAs and HPs, could play a key role in developing standardized protocols for PE based on the analysis of available national standards, relevant literature and data, relevant risk factors that indicate rejection criteria and limitations that are present for deceased donors.

Responses to the HP survey came from a wide range of organizations of different types and sizes, which was reflected in the number of PEs performed by each organization as a whole, and by individuals completing the survey. This broad spread of responses gives a valuable insight into real-life practices. For analysis, responses relating to organ donors were compared with responses relating to tissue and cornea donors, although any clear differences between responses for tissue and cornea donors are also highlighted and discussed. A previous national survey carried out in Australia [8] was targeted at organ and tissue coordinator nurses, while our survey was open to anyone performing PEs. For both organs and tissues/corneas, more than 2/3 of responses were from individuals with a medical background.

When reviewing the responses relating to PE techniques, taking into account all responses, the results of this survey are broadly comparable to the aforementioned Australian survey. However, if the separate responses relating to organ and tissue/cornea donors are considered, there are clear differences: while observation is performed consistently in almost all donors, palpation is done in only 58% of tissue donors compared to 88% of organ donors, and auscultation and percussion are rarely performed for tissue/cornea donors because it does not give relevant information post-mortem. This would explain the observed tendency for PE of tissue/cornea donors to take a shorter amount of time than PE of organ donors (Table 2). There are differences between organ and tissue/cornea donors in the frequency with which different types of examination methods are used (Table 5). This is not surprising, and probably reflects the different circumstances under which PE is performed for these types of donors, such as whether the PE is done pre- or post-mortem, and the number of individuals present to perform PE. For example, where a PE is being performed by a single person, as is common with corneal donors, it is not practical to turn a donor and examine the dorsal surface. Similarly, if PE is performed post-mortem after rigor mortis has set in, as is often the case with tissue and cornea donors, techniques such as opening and examining the oral cavity or palpating the lymph nodes may be impractical. However, even for organ donors, there was no consistent approach regarding the examination methods used. The extent to which these differences lead to differences in the quality and safety of the final organ or tissue remains to be investigated. It should be noted that non-invasive internal scanning performed in organ donors can add value to the PE, but it is not a substitute for visual inspection to note external findings.

Where an abnormal finding was identified during PE, respondents performing PE of organ donors much more frequently reported that they had options for further investigation, such as biopsy for histopathological investigation, other non-invasive examinations such as CT, MRI, or X-ray, or obtaining a second opinion from a colleague (Table 6). The most likely reasons for this difference are the clinical setting in which the PE is performed, and the risk-benefit profile of organ and tissue/cornea transplantation. It was notable that taking of biopsies was less common with cornea donors than with tissue donors. This perhaps reflects the fact that most malignancies are not a contraindication for cornea donation. One retrospective study on potential tissue donors [9] reported that quickly identifying and taking biopsies of suspicious lesions without needing to interpret the findings to determine donor eligibility at the time of procurement could be beneficial for the time management of procurement teams. Of 561 biopsies taken from abnormal findings identified in the PE during the study period (January 2005 to March 2010), the results showed that the tissue did not need to be rejected in 552 (98.4%) cases; the procured tissue from only 9 (1.6%) donors was discarded due to the biopsy results (five for malignancy and four for infection).

In general, the most common abnormal findings reported in PE of tissue donors related to superficial skin findings, such as suspicious injection marks or skin lesions, and for cornea donors, corneal lesions. This is consistent with the PE techniques used for these types of donor, as discussed earlier. It is also consistent with the observations reported in the systematic review [7], where the authors found that almost all articles discussing PE findings that may pose higher risk included findings such as jaundice, tattoos, body piercing, nonmedical injection sites, signs of sexually transmitted infections, scars, oral thrush, and skin lesions, all of which can be identified by visual inspection during a PE.

A significant proportion (37%) of the HP respondents doing PE had not received specific training, higher than was reported in the Australian survey (23%). Of those who received training, 82% felt it was extremely or very valuable. It is important to define the content of training and competency assessment programs taking into consideration the limitations of doing PE after rigor mortis. It is also essential to agree upon the minimum set of physical signs to assess during PE. For example, Van Wijk et al. [10] used a risk assessment-based approach based on the Failure Mode and Effects Analysis model. In their study, 106 signs that could be identified in PE were scored on different criteria, considering available control measures specified in the EU Tissue and Cell Directive [1] or other sources. They proposed risk management procedure to identify minimal necessary content of PE in potential tissue donors and suggested that signs of advanced infection with HIV, hepatitis B/C and syphilis can be omitted, since these contraindications will be detected by the required serological testing. When further defining these issues, the limitations for performing PE should also be taken in consideration, e.g., when only one person is performing the procurement, as is common with cornea donors, and is unable to turn the donor.

Despite the discussed limitations of PE in deceased donors, the majority of respondents (75%) felt that it very or extremely valuable, with the identification of potential medical contraindications to donation and the exclusion of high-risk individuals given as the most important reasons for performing PE. For both organ and tissue/cornea donors, a similar level of importance was accorded to the value of PE for evaluating graft quality, whilst responders for organ donation placed a higher level of importance on the value of PE for preventing donor to recipient disease transmission.

Donor to recipient disease transmission remains a fortunately rare event following organ or tissue transplantation. In EU members states, there is a requirement of establishing a system for the reporting and management of Serious Adverse Reactions and Events imposed by Directive 2010/53/EU, and this is reiterated in the EDQM guides. The Notify Library [11] was established by the World Health Organisation and the Italian National Transplant Centre, with the collaboration of the EU funded project SOHO V&S (Vigilance and Surveillance of Substances of Human Origin and serves to collate reports of adverse events resulting from transplant/transfusion of medical products of human origin. It is imperative that these events are reported and systematically audited.

The limitations of this study must be acknowledged. Firstly, a response to the HA survey was received from only 34 (81%) of the CD-P-TO member and observer countries surveyed. Responses to the HP survey were received from 22 (52%) of the countries surveyed, with some countries submitting multiple responses. The survey was circulated to HPs via CD-P-TO representatives of their countries, therefore responses received may not have included all organisations undertaking PE. The profile of the respondents also varied. The outcomes therefore may not represent a systematic response. It should also be considered that survey was performed to determine the requirement for and practice of PE; it does not attempt to make any determination regarding the best practice of PE*.*

## Conclusion

This is the first survey that has analyzed the differences in the PE between deceased organ and tissue/cornea donors. The HP survey highlighted wide variations in practice and the HA survey demonstrated the absence of international standards in this area. It is likely that the variations in practice demonstrated in this survey are due to discrepancies in training and education, and the lack of standardized guidelines. We strongly suggest that international guidelines be developed to specify the minimum requirements for PE in organ and tissue donors, to be accompanied by appropriate training materials.

Given the limited published literature, it is difficult to determine the added value and effectiveness of PE in contributing to the safety and quality of organs and tissue grafts for clinical use. A risk assessment-based approach, similar to that described by van Wijk et al. [10], could be useful for developing a minimum set of physical assessment criteria, and practical guidelines. More published data relating to the impact of PE on donor deferral would certainly be of value. More importantly, the survey has demonstrated the need to differentiate PE of organ donors from PE of tissue and cornea donors, and to apply a risk-based approach when developing guidance. One size does not fit all!

## Data Availability

The raw data supporting the conclusion of this article will be made available by the authors, without undue reservation.

## Footnotes

^1^The European Committee on Organ Transplantation (CD-P-TO) is the steering committee in charge of organ, tissue and cell donation and transplantation activities at the European Directorate for the Quality of Medicines and Healthcare (EDQM) of the Council of Europe. As of March 2023, the CD-P-TO is composed of 37 members (Albania, Austria, Belgium, Bulgaria, Croatia, Cyprus, Czech Republic, Denmark, Estonia, Finland, France, Germany, Greece, Hungary, Ireland, Italy, Latvia, Lithuania, Luxembourg, Malta, Republic of Moldova, Montenegro, Netherlands, North Macedonia, Norway, Poland, Portugal, Romania, Serbia, Slovak Republic, Slovenia, Spain, Sweden, Switzerland, Turkiye, Ukraine, and the United Kingdom) and 20 observers [Armenia, Canada, Georgia, Israel, United States, Council of Europe Committee on Bioethics, DTI Foundation, European Association of Tissue and Cell Banks, European Commission, European Eye Bank Association, European Society for Blood and Marrow Transplantation, European Society for Organ Transplantation, European Society of Human Reproduction and Embryology, Eurotransplant, Scandiatransplant, South-Europe Alliance for Transplants, The Transplantation Society, United Network for Organ Sharing, World Health Organization (WHO), and the World Marrow Donors Association].
