# Supplementary material for: Physical Examination of Potential Deceased Organ and Tissue Donors: An Overview of the European Landscape
Source: Transpl Int. 2023 Jul 21;36:11394. doi: 10.3389/ti.2023.11394 (PMC10401427; doi:10.3389/ti.2023.11394)
Supplement: Supplementary file 1 [file DataSheet2.pdf]

Physical Examination of potential deceased organ and tissue donors - A survey of current professional practice

Introduction

Physical examination (PE) is used in conjunction with review of medical history obtained from referring professionals, interviews with donor families, information from general practitioners, autopsy reports (if applicable) and screening tests as part of comprehensive donor evaluation.

The EDQM “Guide to the quality and safety of organs for transplantation” and the “Guide to the quality and safety of tissues and cells for human application” provide information on signs to look for during the PE of deceased organ and tissue donors. However, this is based on guidance derived from historical practices and may not reflect advances in technology and understanding. Although the importance of conducting a PE in donor medical assessment is highlighted in the limited number of published articles, there is no strong evidence provided to support this statement. It should be acknowledged that the extent of the PE for organ donors (who may also donate tissues) performed prior to organ donation may vary from the PE that is feasible in deceased tissue-only donors (after refrigeration) performed in the mortuary at the time of tissue retrieval.

The purpose of this survey is to gather information on current practices in the PE of deceased donors in member states of the Council of Europe. This definition includes all types of non-living donors (Donation after Circulatory Death [DCD] controlled/uncontrolled, Donation after Brainstem Death [DBD] & deceased tissue donors). The findings will be used for peer review to develop best practice guidelines to assure the consistency and quality of PE across different member states.

This is a short survey and will take less than 15 minutes to complete.

Physical Examination of potential deceased organ and tissue donors - A survey of current professional practice

Your details

1. Job role (e.g. Donor Coordinator, Medical Director, Transplant Surgeon, etc.)

[ ]

2. Role (select all that apply)

- [ ] Consent
- [ ] Donor evaluation
- [ ] Procurement
- [ ] Processing
- [ ] Transplant
- [ ] Other (please specify below)

Other

[ ]

3. Name of your organisation

[ ]

4. Your country

[ ]

5. Can we contact you if further information is required ?

- ( ) Yes
- ( ) No

6. If answering 'yes' to the previous question, please provide your contact details below

Name: [ ]

Position: [ ]

Email address: [ ]

Telephone No: [ ]

Physical Examination of potential deceased organ and tissue donors - A survey of current professional practice

Organisation details

7. Please describe the type of donor for which you facilitate donation (select one). If you facilitate donation from more than one type of donor group given below, please use a separate questionnaire for each type.

- ◯ Deceased organ donors (who may also donate tissues/corneas)
- ◯ Deceased tissue-only donors (who may also donate corneas)
- ◯ Deceased cornea-only donors

8. What is the role of your organisation in facilitating organ, tissue/cornea donation? (Tick all that apply)

- ☐ Organ/Tissue procurement organisation (donor consent, medical history and procurement)
- ☐ Tissue Establishment (procurement, processing, storage and distribution)
- ☐ Tissue Establishment – procurement by external organisation with service level agreement

9. When is donor PE routinely performed in your organisation/establishment?

- ◯ During/after donor medical assessment (ie: before death in ICU/immediately post mortem but pre-refrigeration): Go to Q10
- ◯ Prior to procurement (after refrigeration): Go to Q10
- ◯ PE is not performed in our establishment (please provide reason(s) in the box below and go to Q32)

Reason(s) for not performing PE

[ ]

10. How many deceased donor PEs were performed during the last calendar year (2019) in your organisation/establishment? (please answer to the best of your knowledge)

[ ]

11. How many Health Care Professionals (physician, surgeon, nurse, donor coordinator, scientist etc.) performed the donor PE during the last calendar year (2019) in your organisation/establishment?

[ ]

12. How many donor PEs did **you** (person completing the questionnaire) perform during the last calendar year? (2019). **If you do not perform PE please indicate this as 0**. Please feel free to request a healthcare professional routinely performing PE in your organisation to complete a questionnaire as well.

13. Please describe the setting where the donor PE is performed: (check all that apply)

- [ ] Hospital setting (intensive care unit/operating theatre)
- [ ] Hospital mortuary
- [ ] Forensic department
- [ ] Other (please specify below)

Other (please specify)

14. Who performs the donor PE in your establishment? (select all that apply)

- [ ] Health care professional in charge of the donor (GP, hospital physician, nurse, etc.)
- [ ] Organ or tissue coordinator
- [ ] Professional from the procurement team of the tissue establishment
- [ ] Pathologist/forensic examiner
- [ ] Other (please specify)

Other (please specify)

15. What are the basic qualifications of the Health Care Professional performing the donor PE in your establishment mentioned in the answer above?

- ○ Medical
- ○ Nursing
- ○ Graduate (e.g. science degree) or similar professional qualifications
- ○ Other (please specify):

Other (please specify)

16. Does your establishment/organisation use total body CT scan as a routine examination for tissue/organ donors?

- Yes (always)
- Yes in selected cases
- No

Additional information

Carrying out a PE

17. On average, how long does it take you to complete a donor PE?

- ◯ Less than 5 minutes
- ◯ 5-15 minutes
- ◯ 16-30 minutes
- ◯ 31-60 minutes
- ◯ More than 60 minutes

18. How many people should be present to perform PE on an individual donor in your organisation?

	No. of people
Organ donors (who may also donate tissues and eyes/corneas)	
Tissue donors (who may also donate eyes/corneas)	
Eye/cornea-only donors	

19. Which techniques do you use during the donor PE? Tick all that apply. If not relevant or not applicable (e.g. some options may not be applicable to deceased donors), **mark N/A**

	Performed?
Observation	
Auscultation	
Palpation	
Percussion	

20. When donor PE is performed, do you:

	Select appropriate option from drop down menu
Open & examine the oral cavity	
Inspect/examine the genital area	
Turn the donor to examine the back	
Palpate the lymph nodes	
Palpate the breast tissue	
Palpate the abdomen	
Check for evidence of intravenous drug use	

21. What options are available to you in your practice if you identify an abnormal finding? (tick all that apply)

- ☐ Document the findings and proceed/stop
- ☐ Ask a colleague to examine the donor for a second opinion
- ☐ Phone a senior colleague from your team and describe your findings to obtain advice
- ☐ Take a photograph and send it to an external expert (e.g. skin specialist)
- ☐ Take a biopsy for histopathology examination
- ☐ Other tests or non-invasive examinations (CT, MRI, X-ray)
- ☐ Review medical notes and/or contact General Practitioner
- ☐ Other (please provide details in the box below)

Other (Additional information)

22. Have you or your colleagues ever identified an abnormality during a PE that stopped donation from going ahead at that point or that had resulted in subsequent rejection of the organs/tissues?

- ○ Yes (please provide brief details in the box below)
- ○ No

Further information

23. What are the most common findings (list up to 3) during the PE that are relevant in donor screening because it might lead to a contraindication in combination with other information? such as medical notes/test results)?

1.

2.

3.

24. What are the top 3 findings during PE alone that would be considered as contraindications to exclude the donor where that information is unknown before the PE (e.g. venepuncture marks) ?

1.

2.

3.

25. For deceased tissue & cornea donors only : Which common issues in your practice are barriers to performing a detailed PE (select all that apply).

For organ donors, please select the N/A option

- ☐ Performing PE alone
- ☐ Ethical issues connected with genital examination
- ☐ Lack of experience
- ☐ Lack of knowledge
- ☐ Rigor mortis
- ☐ Inadequate facilities/lighting
- ☐ Time constraints
- ☐ Other (please specify)
- ☐ N/A

Training

26. Have you received any specific training in how to perform a donor PE for organ/tissue/eye donors?

- ◯ Yes
- ◯ No (proceed to Q32)

27. If you have received training, when did this take place?

- ☐ During my degree studies
- ☐ Provided by external bodies outside the organisation
- ☐ Before starting to work in my establishment (during induction including theory)
- ☐ Case-by-case training by another colleague during my working practice
- ☐ Other - please specify below

Additional information

28. How was the training delivered? (Tick all that apply)

- ☐ Reading the SOP
- ☐ PowerPoint presentation
- ☐ eLearning course
- ☐ Practical simulation
- ☐ Other (please specify)

Other (please specify)

29. Did the training include how to document PE findings?

- ◯ Yes (please include details in the comment box)
- ◯ No

Further details

30. How would you describe the value of the training for your daily work?

- ◯ Extremely valuable
- ◯ Very valuable
- ◯ Moderately valuable
- ◯ Slightly valuable
- ◯ Not valuable at all

31. How do you maintain your competency? (Please tick all that apply)

- ☐ Training updates (How often? Please provide details in the comment box)
- ☐ Audit
- ☐ Peer-review practice
- ☐ Task-based training using SOPs
- ☐ Other (please specify)

Further details

General opinions of PE

32. In your opinion, what is the value of the PE in the evaluation of deceased donors? Why? Please provide a reason for your answer in the comment box.

- ◯ Extremely valuable
- ◯ Very valuable
- ◯ Moderately valuable
- ◯ Slightly valuable
- ◯ Not valuable at all

Reason for your answer

33. What do you think are the top 3 most important reasons for doing a PE prior to organ and tissue/cornea donation? Please explain your answer in the comment box below.

- ☐ To identify the cause of death
- ☐ To identify potential medical contraindications
- ☐ To exclude high-risk individuals (e.g. social risks)
- ☐ To confirm information available from other sources
- ☐ To comply with regulations and guidelines
- ☐ Transplant centres are interested in the donor PE
- ☐ Not important, as the PE is of limited value for tissue donors including eye donors

Reason for your answer

34. On a scale of 1-10, with 1 indicating no importance and 10 indicating extreme importance, what is the value of abnormal findings in the donor PEs in prevention of donor-recipient disease transmission (safety) or graft quality:

	1	2	3	4	5	6	7	8	9	10
Tissue/Cornea donors: Donor-recipient transmission	◯	◯	◯	◯	◯	◯	◯	◯	◯	◯
Tissue/Cornea donors: Graft Quality	◯	◯	◯	◯	◯	◯	◯	◯	◯	◯
Organ donors: Donor-recipient transmission	◯	◯	◯	◯	◯	◯	◯	◯	◯	◯
Organ donors: Graft Quality	◯	◯	◯	◯	◯	◯	◯	◯	◯	◯

35. Thank you for completing the survey. If you have any further comments, please use the box below
