# Supplementary material for: Physical Examination of Potential Deceased Organ and Tissue Donors: An Overview of the European Landscape
Source: Transpl Int. 2023 Jul 21;36:11394. doi: 10.3389/ti.2023.11394 (PMC10401427; doi:10.3389/ti.2023.11394)
Supplement: Supplementary file 2 [file DataSheet1.pdf]

Introduction

Physical examination (PE) is used in conjunction with review of medical history obtained from referring professionals, interviews with donor families, information from general practitioners, autopsy reports (if applicable) and screening tests, as part of comprehensive donor evaluation.

The EDQM "Guide to the quality and safety of organ for transplantation" and the "Guide to the quality and safety of tissues and cells for human application" provide some information on signs to look for in the PE of deceased organs and tissue donors. However, this is based on guidance derived from historical practices and may not reflect advances in technology and understanding. Although the importance of conducting a PE in donor medical assessment is highlighted in the limited number of published articles, there is no strong evidence provided to support this statement. It should be acknowledged that the options to perform the PE of organs donors (who may also donate tissues) prior to organ procurement are different from those of the PE that is feasible in deceased only tissue donors (after refrigeration) and performed in the mortuary at the time of tissue retrieval.

The purpose of this survey is to gather information regarding national regulations and guidance documents in member states of the Council of Europe. A separate survey will be sent to organ/tissue procurement organizations and tissue establishments to gather information on current practice.

Your details

1. Name of the organisation

2. Your country

3. Job title/role of person completing the survey

4. Can we contact you if further information is required ? (If answering 'Yes' please provide your contact details below)

- Yes
- No

5. Contact details (optional)

Name:

Email address:

Telephone No:

Questionnaire

6. In your country, a Physical Examination (PE):

- [ ] Is recommended in organ donors
- [ ] Is mandatory in organ donors
- [ ] Is recommended in tissue donors
- [ ] Is mandatory in tissue donors
- [ ] Is recommended in both organ and tissue donors
- [ ] Is mandatory in both organ and tissue donors

7. In your country, do you have national regulations related to the PE of deceased donors?

- ( ) Yes
- ( ) No
- ( ) I don't know

If answering yes, please provide a link to the national regulation

8. In your country, do you have national guidance documents related to the PE of deceased donors?

- ( ) Yes
- ( ) No
- ( ) I don't know

If answering yes, please provide a link to the national guidance

9. In your country, do you have a uniform template/model (forms) to collect the findings of the PE of deceased donors?

- ( ) Yes, for organ and tissue donors
- ( ) Yes, only for organ donors
- ( ) Yes, only for tissue donors
- ( ) No

10. In your country, are there specific training courses which cover aspects related to the PE of tissue donors?

- ◯ Yes
- ◯ No
- ◯ I don't know

11. Please use the box below to provide any further comments
